# Upholding academic freedom: a call to protect freedom of expression and science in Ecuador and beyond

**DOI:** 10.3389/fpubh.2023.1259110

**Published:** 2023-09-07

**Authors:** Esteban Ortiz-Prado, Juan S. Izquierdo-Condoy, Jorge Vasconez-Gonzalez, Miguel Angel Garcia-Bereguiain

**Affiliations:** One Health Research Group, Faculty of Medicine, Universidad de Las Américas, Quito, Ecuador

**Keywords:** academic freedom, freedom of expression, science, low income, Ecuador

We are writing to express our concern regarding the challenges to freedom of expression and scientific advances currently experienced by public health professionals in Ecuador.

Freedom of expression, a human right enshrined in most democratic nations since the last century, is vital for empowering individuals to openly share data, information, suggestions, and constructive criticism about their governments and organizations. While freedom of expression is not an absolute right and may be subject to certain limitations and regulations, public health professionals must be familiar with their organization's policies and procedures concerning communication and expression of opinions in the workplace. Employers, in turn, should foster a respectful and inclusive work environment that values diverse opinions and encourages constructive dialogue and debate.

It is well-established that science and innovation are essential drivers for the progress and development of nations and need to be supported particularly in low- and middle-income countries (LMICs). Nonetheless, scientific advances often encounter resistance not only from academic groups but also from governments and communities. It is crucial to foster and safeguard academics, particularly in fields where innovation and creativity are paramount (1), without fear of discrimination or retaliation from authority figures.

Regrettably, in numerous countries, public health professionals or university faculty, in general, face pressures and reprisals related to job stability, hindering their ability to freely express their opinions (2). In the realm of health sciences, it is not uncommon to hear accounts from physicians unable to publicly discuss resource or supply shortages in hospitals or from researchers unable to publish their findings due to political interference, particularly in the context of democratic LMICs.

We wish to express our deep concern about the recent encroachments on freedom of expression and scientific thought in Ecuador. It is troubling to learn that doctors in public hospitals cannot voice concerns about insufficient medicines or supplies due to pressure from authorities dictating what can and cannot be expressed about this situation, even when those concerns are properly addressed not only in mass media but also in specialized public health journals. For instance, it is unacceptable that health professionals could not discuss the limitations they face in their work practices due to inadequate personal protective equipment and safety measures during the COVID-19 pandemic. This constitutes a clear violation of the right to freedom of expression and a challenge for scientific advances.

Moreover, there was a recent striking case of persecution involving members of the main Ecuadorian public health who challenged academic freedom in Ecuador. These individuals sought to publish research on the region's first reported case of H5N1 influenza infection, which, despite exposing weaknesses in the epidemiological surveillance and control system, provides valuable information for authorities to implement control and surveillance programs for a new issue (3). The impact of this publication made one of the main newspapers in Spanish (“El Pais”) publish a report about this study. By contrast, mid-level authorities from the Ministry of Health in Ecuador started a disciplinary process against some of the authors of this study with unfounded accusations of ethical misconduct. They never addressed neither the scientific journal with any concerns nor the universities involved in the study. Moreover, in their disciplinary report, they made the authors responsible for the report in the newspaper, made by an independent journalist. The professional misconduct and bias in this fake disciplinary were finally corrected by superior authorities from the Ministry of Health after an active social network debate. The fact that they were persecuted for simply sharing scientific knowledge without any conflict of interest is a blatant violation of their right to freedom of expression and scientific thought, no matter whether there was a happy end to this story.

In light of these developments, we urge the Ecuadorian Public Health authorities to recognize the importance of protecting and promoting freedom of expression, scientific advances, and academic freedom. It is essential to create a society of openness and transparency that encourages citizens to express scientific knowledge freely and without fear of reprisals or repercussions of any kind, and public health practice cannot be excluded from that. Moreover, science must provide the necessary foundation for informed decision-making by the authorities in public health policy and beyond. Considering the incalculable consequences of silencing scientists and the numerous challenges faced by science in Ecuador and other LMICs, progress and social development depend on securing academic freedom (4, 5).

In conclusion, we call on the Ecuadorian government to promote actions to protect the right to freedom of expression and support scientific advances. Ensuring that individuals can express their opinions freely without fear of retaliation, and that scientific knowledge can be shared without fear of persecution, is vital for promoting the progress and development of any nation.

## Author contributions

EO-P: Conceptualization, Formal analysis, Investigation, Methodology, Project administration, Writing—original draft, Writing—review and editing. JI-C: Investigation, Methodology, Validation, Writing—original draft, Writing—review and editing. JV-G: Formal analysis, Investigation, Methodology, Writing—original draft. MG-B: Conceptualization, Formal analysis, Investigation, Resources, Supervision, Writing—original draft, Writing—review and editing.
